# Remineralization of enamel subsurface lesions using toothpaste containing tricalcium phosphate and fluoride: an in vitro µCT analysis

**DOI:** 10.1186/s12903-020-01286-1

**Published:** 2020-10-27

**Authors:** Hidenori Hamba, Keiki Nakamura, Toru Nikaido, Junji Tagami, Takashi Muramatsu

**Affiliations:** 1 Department of Operative Dentistry, Cariology and Pulp Biology, Tokyo Dental College, 2-9-18, Kanda-Misakicho, Chiyoda-ku, Tokyo 101-0061 Japan; 2 Cariology and Operative Dentistry, Department of Restorative Sciences, Graduate School of Medical and Dental Sciences, Tokyo Medical and Dental University, Tokyo, Japan; 3 Department of Operative Dentistry, Division of Oral Functional Science and Rehabilitation, School of Dentistry, Asahi University, Mizuho, Japan

**Keywords:** Micro-computed tomography, Enamel subsurface lesions, Mineral density, Remineralization, Functionalized tricalcium phosphate, Sodium fluoride

## Abstract

**Background:**

This study aimed to compare the efficacies of experimental toothpastes containing functionalized tricalcium phosphate (fTCP) with and without fluoride for in vitro enamel remineralization under pH-cycling conditions.

**Methods:**

To create artificial white spot lesions, 36 bovine enamel specimens were immersed in a demineralization solution for 10 days. During pH-cycling for 12 days, the specimens were divided into four groups based on the experimental toothpaste type used: (a) fTCP-free, fluoride-free (fTCP − F −); (b) fTCP-containing, fluoride-free (fTCP + F −); (c) fTCP-free, fluoride-containing (fTCP − F +); and (d) fTCP-containing, fluoride-containing (fTCP + F +). Micro-focus X-ray computed tomography (μCT) scans of all specimens were obtained before demineralization, after demineralization, and after pH-cycling. The mineral density and mineral loss (ΔZ) in the enamel subsurface lesions were measured and the percentage of remineralization (%R) was calculated from ΔZ after demineralization and pH-cycling. One-way ANOVA with Tukey’s test was used for statistical analysis of the %R values. The treated enamel surface was investigated via scanning electron microscopy (SEM).

**Results:**

The fTCP − F − group presented with the lowest amount of mineral gain after pH-cycling. In contrast, the fTCP + F + group showed the highest degree of remineralization within all lesion parts. The %R was highest in the fTCP + F + group (38.2 ± 7.8, all *P* < 0.01). SEM revealed the presence of small crystals on the enamel rods in the fTCP + F − and fTCP + F + groups.

**Conclusions:**

The experimental toothpaste containing fTCP and fluoride increased remineralization of the artificial enamel subsurface lesions during pH-cycling. Furthermore, fTCP and fluoride appear to act independently on the remineralization of enamel subsurface lesions, although they coexisted in one toothpaste type.

**Trial registration:** This is not a human subject research.

## Backgrounds

Dental caries is a multifactorial disease caused by the damaging effect of acids on the enamel surface [1, 2]. The enamel is a relatively stable structure characterized by a dynamic balance between demineralization and remineralization [3, 4]. However, a disruption in this balance can lead to the development of demineralized lesions in the enamel. Remineralization is a repair mechanism that occurs naturally in tooth lesions. In this process, the demineralized tooth areas depleted of crystals are deposited with plaque/salivary calcium and phosphate ions, resulting in net mineral gain. Free F^−^ ions present in the oral environment can facilitate the deposition of calcium and phosphate ions into the crystal lattice and the resultant formation of fluorapatite, which is notably resistant to any subsequent acid action [5]. Dental caries is a disease continuum that starts with the loss of ions from apatite crystals in the early stage, leading to lesion cavitation [6]. Early stopping or reversing the formation of demineralized lesions should be the principal goal to prevent the risk of cavitation and the subsequent need for any invasive interventions [5, 7–9].

White spot lesion (WSL), with a characteristic intact external surface and demineralized subsurface, is clinically considered the first sign of dental caries [10]. Under appropriate conditions, WSLs can be reversed, and various approaches have been suggested for the early therapeutic management of WSLs [11, 12]. To date, the majority of treatments have been based on remineralization, mainly using fluoride [12, 13].

Fluoride is a well-known remineralizing agent that mingles with oral fluids at the enamel interface and subsurface lesions present on the teeth and reacts with calcium and phosphate ions to generate fluorapatite.

Beta-tricalcium phosphate (β-TCP) is an attractive calcium phosphate system as it can emerge as a transitional phase in the conversion of hydroxyapatite [14]. It is compatible with biological systems and is bioactive in nature [15, 16] as well as displays lattice defects that help in crystal modification [17]. In a precious study, the structure of β-TCP was altered by combining it with carboxylic acids and surfactants to generate functionalized β-TCP (fTCP) [18]. The function of fTCP is to block premature interactions between fluoride and calcium, thereby allowing the formation of a targeted low-dose fluoride delivery system when applied using dentifrices or mouthwashes [19]. The primary purpose of fTCP is to improve the action of fluoride on the tooth surface, whereas remineralization is mostly driven by salivary calcium and phosphate ions. The protective effect of fTCP-containing toothpastes on the demineralization of the enamel has been demonstrated by studies using the microhardness test [18, 20–24], confocal microscopy [25], and quantitative light-induced fluorescence [26] of the enamel. Moreover, fTCP has been demonstrated to exhibit significant remineralizing effects on the enamel surface [22]. However, there have been very few reports to date regarding its effect, either alone or in combination with fluoride, on the mineral changes in enamel subsurface lesions. To the best of our knowledge, there are no studies on the effect of fTCP and fluoride on the mineral changes in enamel subsurface lesions.

Some non-destructive techniques allow the long-term evaluation of the effects of remineralizing agents on the enamel and dentin [27–30]. Micro-focus X-ray computed tomography (μCT) is useful for evaluating the mineral density (MD) and mineral structure of bones, teeth, and similar tissues without causing tissue destruction [4, 31, 32]. We previously reported that μCT-based measurements with appropriate correction for the beam-hardening effect can help in assessing the in vitro demineralization and remineralization of WSL in teeth [33].

Therefore, this study aimed to assess the efficacy of experimental toothpastes containing fTCP and fluoride for enamel remineralization under pH-cycling conditions using μCT. We hypothesized that toothpastes containing fTCP and fluoride have a better remineralization effect compared with those without fTCP or fluoride.

## Methods

### Tooth preparation

**Fig. 1:**
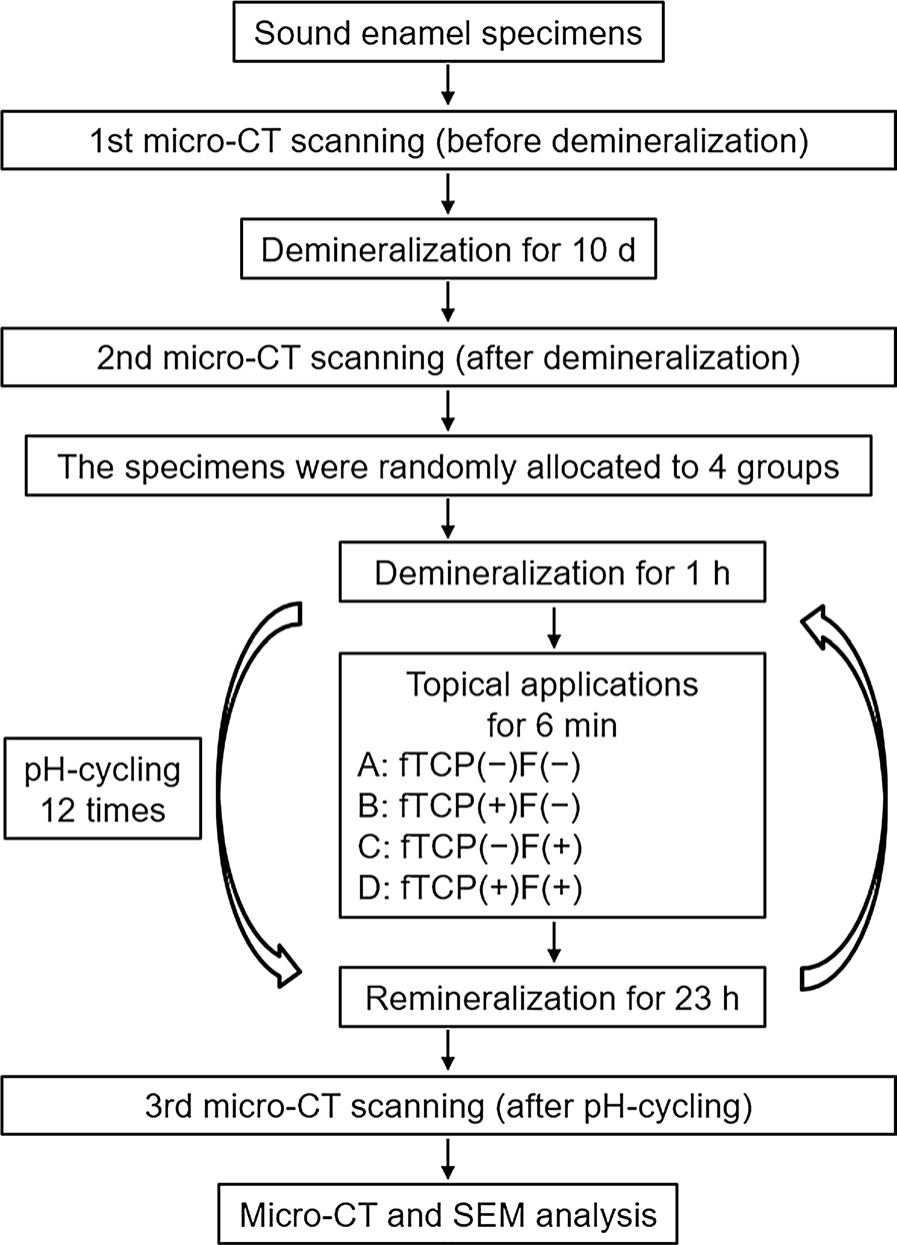
Flow chart of the experimental design of the study

A procedural flow chart of the present study is shown in Fig. 1. Thirty-six extracted, permanent bovine incisors without damage were stored under freezing conditions until further use. All adherent soft tissues on the teeth were removed by thoroughly cleaning and washing the teeth under running water. A low-speed diamond saw (Isomet, Buehler, Lake Bluff, IL, USA) was used under water to remove the root of the tooth with extra care to keep only the crown. Specimens were prepared by cutting the teeth into 4 × 4 × 3 mm enamel-dentin blocks using the low-speed diamond saw under water. The enamel surfaces were ground flat using 600- to 2000-grit silicon carbide papers (Fuji Star, Sankyo Rikagaku, Saitama, Japan) under water. To set a reference landmark for the μCT scans, a hole (1.0 mm in diameter, 0.5 mm in depth) was made at the side of the tooth using a diamond bur (440SS ISO # 010, Shofu, Kyoto, Japan). Then, nail polish (680, Revlon, New York, NY, USA) was used to cover the surfaces, leaving a window (2 × 2 mm) to expose the specimen’s polished enamel surface.

### Lesion formation

Subsurface enamel lesions were produced in the enamel using the method described by Margolis et al.[34]. Each specimen was then separately immersed in 10 mL of a demineralization solution (17.8 mM CaCl_2_, 8.8 mM KH_2_PO_4_, 100 mM lactic acid, and 1.0 mM NaN_3_; pH was adjusted to 4.3 using 10 mM KOH) at 37 °C for 7 days [34]. The demineralization solution was refreshed daily. To align the demineralization conditions, 36 specimens were selected with initial demineralization within the standard deviation (SD) of the mean demineralization.

### pH-cycling and treatment with toothpaste

After lesion formation, the specimens were coded and randomly divided into four experimental groups based on the type of paste used (n = 9 for each group): (a) fTCP-free, fluoride-free toothpaste (fTCP − F −); (b) fTCP-containing, fluoride-free toothpaste (fTCP + F −); (c) fTCP-free, fluoride-containing toothpaste (fTCP − F +); and (d) fTCP-containing, fluoride-containing toothpaste (fTCP + F +) (Experimental paste, 3M, Tokyo, Japan). Table 1 shows the details of the provided materials. After demineralization, the specimens were removed from the solution and subjected to μCT scanning.

**Table 1:** The materials used in this study and their composition

Code	Material	Composition	Manufacturer
fTCP − F −	Experimental paste	fTCP-free, NaF-free Silica-based paste: water, sorbitol, silicon dioxide, glycerin, polyethylene glycols, sodium lauryl sulfate, titanium dioxide, isopropyl methyl phenol, carboxymethyl cellulose sodium, saccharin sodium	3M, Tokyo, Japan
fTCP + F −	Experimental paste	Silica-based paste with fTCP without NaF	3M, Tokyo, Japan
fTCP − F +	Experimental paste	Silica-based paste with 950 ppmF as NaF without fTCP	3M, Tokyo, Japan
fTCP + F +	Experimental paste	Silica-based paste with 950 ppm F as NaF and fTCP	3M, Tokyo, Japan

*fTCP* functionalized tricalcium phosphate, *F* fluoride, *NaF* sodium fluoride

All specimens were treated to a standard regime of pH-cycling [35]. The enamel blocks were alternately immersed in remineralization solution (1.5 mM CaCl_2_, 0.9 mM KH_2_PO_4_, 130 mM KCl, and 20 mM HEPES; the pH was adjusted to 7.0 with 10 mM KOH and 1.0 mM NaN_3_) and demineralization solution (17.8 mM CaCl_2_, 8.8 mM KH_2_PO_4_, and 100 mM lactic acid, 1.0 mM NaN_3_; pH was adjusted to 4.3 with 10 mM KOH). During each 24-h period, the specimens were immersed in the remineralization solution (4 mL per block) for 23 h and then in the demineralization solution (4 mL per block) for 1 h. The blocks were rinsed with pure water between solution changes. The blocks were treated with the four types of experimental pastes during pH-cycling. Treatments were conducted once each day and one time after demineralization and before remineralization. During each treatment, the specimens were immersed in each treatment solution kept at 37 °C for 6 min, once a day, followed by rinsing with pure water for 1 min, and then finally incubated at 37 °C in pure water. Experimental paste suspensions were prepared at 1:3 dilutions (paste: deionized water) to minimize the influence of other ingredients (e.g., thickeners or ora-base), thoroughly mixed, and subjected to mechanical agitation for 1 min using a vortex mixer (MF-71, TGK, Tokyo, Japan), as described previously [31]. In this treatment, the toothpastes were applied passively by immersion into paste suspensions. pH-cycling and the treatment cycle were continued for 12 days using freshly prepared suspensions each day [36]. After completing pH-cycling and the treatment cycle, the specimens were subjected to μCT scanning for evaluation.

### μCT scanning

A μCT system (inspeXio SMX-100CT; Shimadzu, Kyoto, Japan) was used to assess the changes in MD in the specimens after demineralization and remineralization. Each specimen was mounted on to a computer-controlled turntable, with the treated dentin surface perpendicular to the X-ray beam. A wet wiper roll was placed on top of the specimen to prevent it from drying out during scanning. A 0.2-mm-thick brass filter was used in the beam path to reduce the beam-hardening effect [31]. The tube voltage was set to 100 kV, and a current of 70 µA was applied. The distance between the X-ray source and the specimen was 68.2 mm and that between the X-ray source and the detector was 300 mm. The specimen was rotated 360° in increments of 0.3°. Air calibration of the detector was performed before each scanning to minimize the number of ring artifacts. Additionally, eight-frame averaging was applied during the acquisition phase to improve the signal-to-noise ratio [37]. The 360° rotation of each specimen was performed at an integration time of 6 min. Data were acquired as 250 TIFF files to reconstruct the three-dimensional (3D) images of the coronal aspect at a resolution of 1024 × 1024 pixels and an isotropic voxel size of 7.0 μm. A series of mineral reference phantoms were scanned for MD calibration; these included three hydroxyapatite (HAp) disks (Phantoms; Ratoc System Engineering, Tokyo, Japan) with different concentrations (0.20, 0.40, 0.50, and 0.70 gHAp cm^−3^) of hydroxyapatite crystals embedded in an epoxy resin as well as a pure HAp disk (concentration, 3.14 gHAp cm^−3^; Cellyard; HOYA Technosurgical Corporation, Tokyo, Japan) [33]. Each specimen was subjected to μCT scanning three times within the experimental period as follows: before demineralization (baseline), after demineralization, and after remineralization.

### μCT image analysis

A 3D analysis software (TRI/3D-BON; Ratoc System Engineering) was used to reconstruct 3D images from two-dimensional (2D) images. Grayscale values were converted to MD values (gHAp cm^−3^) using a linear calibration curve based on the grayscale values received from the phantoms (in linear regression, R^2^ > 0.9997). The 3D data images of each treatment from the same specimen were aligned and registered into one coordinate system to compare the changes in the enamel lesions. The rendered 3D volumes were translated and rotated in an optional software (TRI/3D-DIF; Ratoc System Engineering) to visually match the baseline image, which served as the reference. The features used for the corresponding process were sound surface, specimen edges, and the reference landmark on the side of the specimen (the small hole made by a high-speed round diamond bur).

**Fig. 2:**
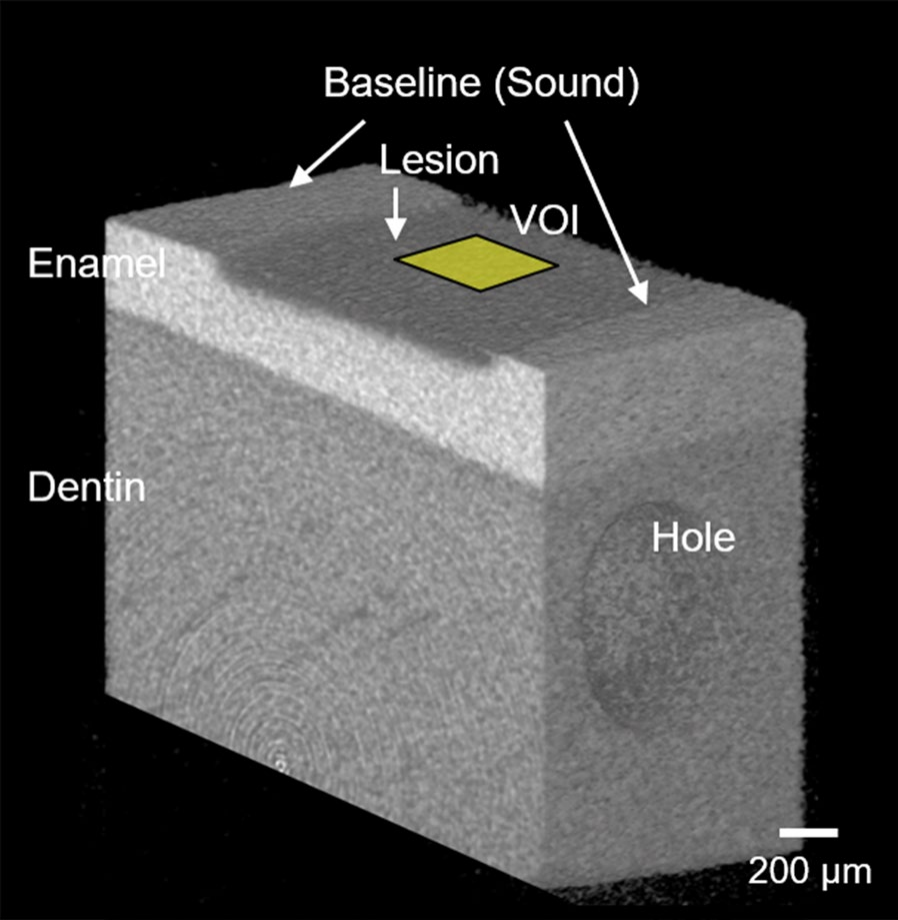
A three-dimensional (3D) micro-focus X-ray computed tomography (µCT) image of the specimen after demineralization. A hole was prepared as a reference landmark. VOI, volume of interest (425 × 425 × 900 µm^3^)

Mean MD values were calculated and plotted against the depth in a volume of interest measuring 425 × 425 × 900 μm^3^ at the center of the test window using an optional software (TRI/TMD; Ratoc System Engineering) (Fig. 2). The MD profile was converted to a relative MD profile by assuming sound enamel with a maximum MD of 100 vol%. Mineral loss (ΔZ; vol% in micrometers) was calculated from the relative MD profiles; the reference point of the depth axis (0 µm) was set at the axial position of the apparent surface of the enamel lesion. To calculate the ΔZ value for each specimen from the profiles, the area under the curve was subtracted from the assumed area of the sound enamel before demineralization. The mean percentage of remineralization (%R) was calculated via trapezoidal integration, according to the following formula:

$$\% {\text{R }} = \, \left[ {\left( {\Delta {\text{Zd }} - \, \Delta {\text{Zr}}} \right)/\left( {\Delta {\text{Zd }} \times { 1}00} \right)} \right]$$

where ΔZd is the difference between the profiles of the area under the sound enamel and the demineralized enamel and ΔZr is the difference between the profiles of area under the sound enamel and the remineralized enamel [38]. These calculations were conducted by importing the MD data into a spreadsheet (Microsoft Excel; Microsoft, Redmond, WA, USA).

### Scanning electron microscopy (SEM) observation

The specimens were prepared as described for μCT scanning. The conditioned enamel surfaces from each group were observed under a scanning electron microscope (JSM-5310LV, JEOL, Tokyo, Japan) after pH-cycling. The specimens were placed on a filter paper placed in a covered glass vial for 24 h at room temperature for desiccation. After gold-sputter coating (SC-701AT, Elionix, Tokyo, Japan), the conditioned specimens were longitudinally fractured at the center using a cutting plier, and the enamel structure was observed cross-sectionally using a scanning electron microscope (JSM-5310LV) at 2,000 magnification (n = 2).

### Statistical analysis

%R was analyzed using one-way analysis of variance (ANOVA) to test the effects of the treatment groups. The Tukey’s test was used for multiple comparisons at the 95% level of confidence. All statistical tests were performed using the SPSS software ver. 22 (IBM, Chicago, IL, USA). *P* values < 0.05 were considered statistically significant.

## Results

### µCT analysis

**Fig. 3:**
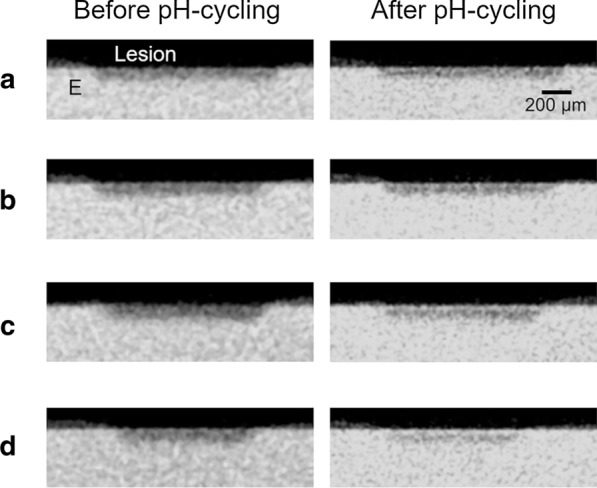
Two-dimensional (2D) micro-focus X-ray computed tomography (µCT) images of single specimens from each group. 2D images of the fTCP − F − (**a**), fTCP + F − (**b**), fTCP − F + (**c**), and fTCP + F + (**d**) groups are shown before and after 12 days of pH-cycling. **e** Enamel

The typical 2D images of the treatment groups after demineralization and pH-cycling are shown in Fig. 3. After remineralization, the specimens in the treatment groups showed mineral recovery in the enamel subsurface lesions. The parts beneath the enamel surface were demineralized in the fTCP − F − and fTCP + F − groups compared with in the fTCP − F + and fTCP + F + groups. The fTCP + F + group showed mineral recovery in most enamel subsurface lesions.

### Mean MD profiles

**Fig. 4:**
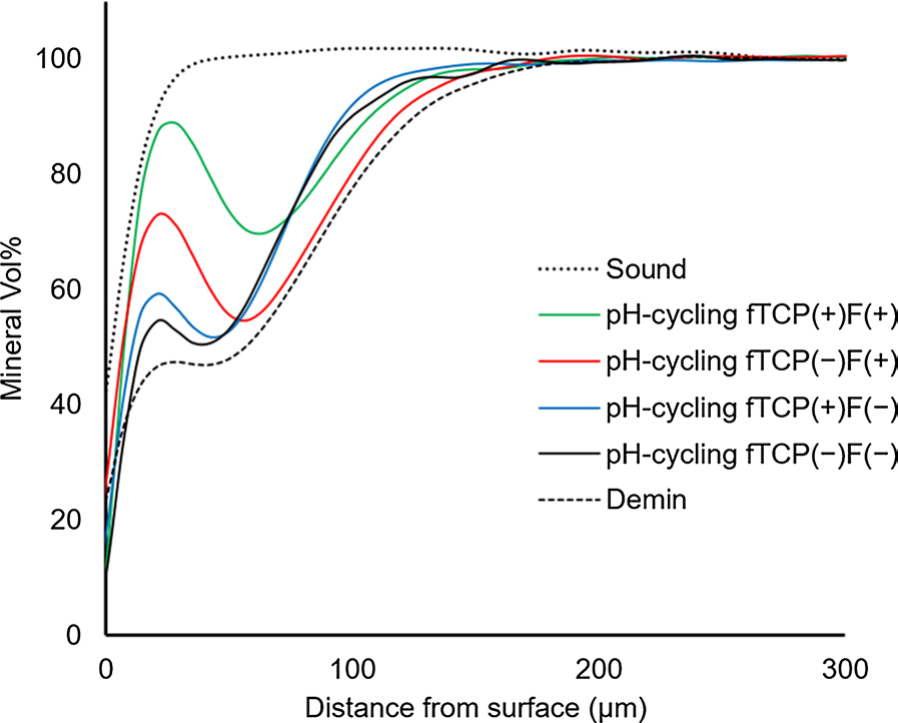
Mean mineral density (MD) profiles of all specimens from the experimental groups (line traced from a composite of all seven in each group). The mean MD profiles of the fTCP − F − , fTCP + F − , fTCP − F + , and fTCP + F + groups are shown. Graphs show the mean MD profiles, as determined via mineral volume (%; y-axis) vs. distance (µm; x-axis) at baseline (sound) and before and after 12 days of pH-cycling

The MD profiles of the enamel in each treatment group are summarized in Fig. 4. After demineralization, all test groups demonstrated a demineralized enamel thickness of approximately 180 µm. After pH-cycling, the fTCP − F − group had the lowest amount of mineral gain. In contrast, the fTCP + F + group showed the highest degree of remineralization within all lesion parts. The surface MD values (around 25 µm) were higher in the fTCP − F + and fTCP + F + groups than in the other two groups. Alternatively, the MD values at the bottom of the specimens (around 150 µm) were higher in the fTCP + F − and fTCP + F + groups than in the other two groups.

### Mean percentage of remineralization

**Fig. 5:**
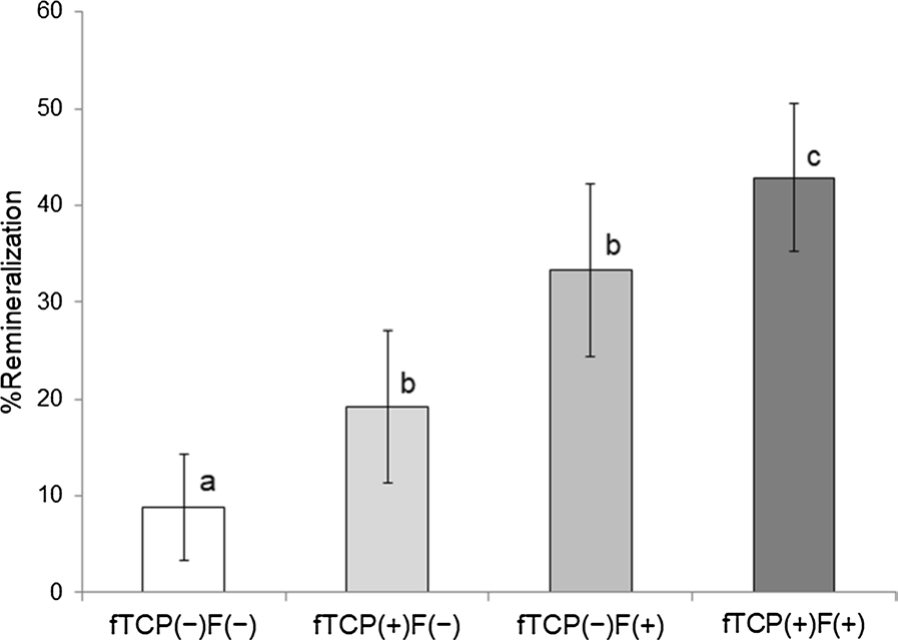
Mean percentage of remineralization in each group. The data are presented as means and standard deviations. Similar lower case letters indicate no statistically significant difference among the compared values (P > 0.05). fTCP, Functionalized tricalcium phosphate; F, Fluoride

The mean %R in each group is summarized in Fig. 5. The mean %R ± SD values were 8.9% ± 5.5%, 19.2% ± 7.9%, 25.3% ± 6.9%, and 38.2% ± 7.8% for the fTCP − F − , fTCP + F − , fTCP − F + , and fTCP + F + groups, respectively, in ascending order. Statistical analysis conducted using one-way ANOVA revealed significant differences between the treatment groups (P < 0.001). In addition, multiple comparisons using the Tukey’s post-hoc test revealed significant differences in %R within the treatment groups. There were significant differences in all groups (P < 0.05, for all comparisons) except between the fTCP + F − and fTCP − F + groups (P = 0.284).

### SEM observation

**Fig. 6:**
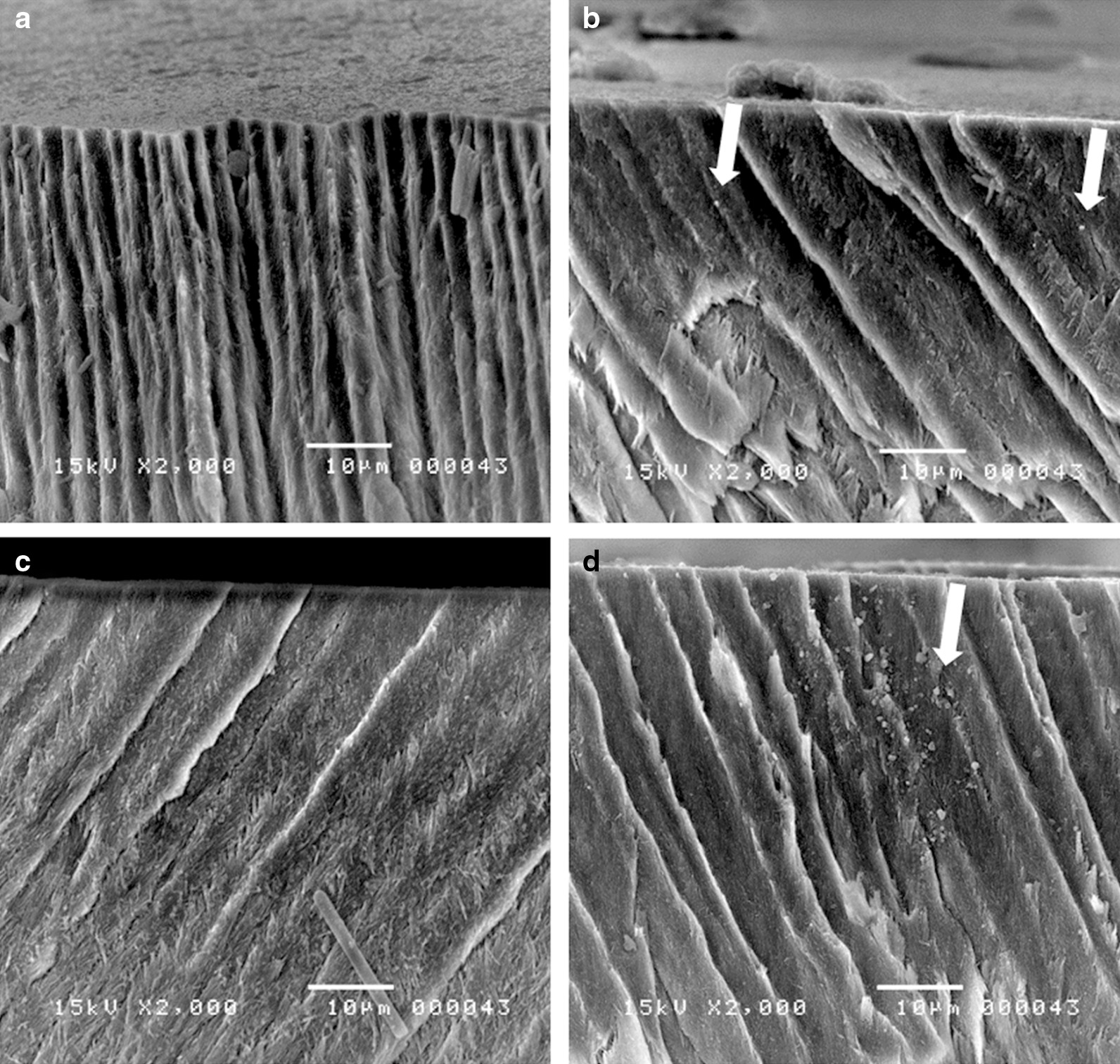
Cross-sectional scanning electron microscopic micrographs of the enamel specimens of the fTCP − F − (**a**), fTCP + F − (**b**), fTCP − F + (**c**), and fTCP + F + (**d**) groups after pH-cycling. Arrows indicate particle deposition on the enamel rods

Representative SEM micrographs of the enamel surfaces after pH-cycling are shown in Fig. 6. In all the groups, the trabecular structures of the enamel appeared as irregular and black areas beneath the surface. SEM analysis revealed that the fTCP − F − group had the narrowest enamel trabeculae among all the tested groups, with dark areas between the trabeculae (Fig. 6a). On the contrary, SEM analysis revealed that the dark area present between the enamel trabeculae was not clear in the fTCP + F − and fTCP + F + groups compared with in the fTCP − F − and fTCP + F − group (Fig. 6b, c). Moreover, SEM analysis revealed the presence of ~ 1-µm-diameter crystal-like structures in the fTCP + F − group (Fig. 6b) and the presence of crystal-like structures with a slightly larger size (diameter of 1–1.5 µm) in the fTCP + F + group (Fig. 6d).

## Discussion

In the present in vitro study, toothpastes with or without fluoride were used to evaluate their efficacies on enamel remineralization. The materials underwent pH-cycling to simulate the saliva and diet cycle. Experimental pastes are designed as daily oral care products to prevent the development of caries, periodontal disease, or acid erosion. In general, toothpastes are recognized as the best source of fluoride, which is known to be most effective against caries in both deciduous and permanent teeth. The concentration of fluoride in the saliva is associated with caries prevention. Furthermore, experimental toothpastes are normally diluted during the process of tooth brushing [39]; therefore, the test pastes used in the present study were diluted threefold with water and were used to simulate oral conditions.

The fTCP + F + group showed the highest degree of remineralization within all lesions parts (Fig. 4). Moreover, the %R of the fTCP + F + group was significantly higher than that of the fTCP + F − group (P < 0.01; Fig. 5). These results are similar to those of a previous in situ study, which demonstrated that the combination of fTCP and fluoride can act together with fluoride to result in significant remineralization [40]. The surface MD values were higher in the fTCP − F + and fTCP + F + groups than in the other two groups, suggesting that fluoride may have had some effect on the enamel surface. The increase in the bottom MD values in the fTCP + F − and fTCP + F + groups compared with in the other two groups may be due to the action of fTCP in the deeper layers. These results reflect the occurrence of remineralization on the surface and deep within the lesion.

fTCP containing sodium lauryl sulfate or fumaric acid is intended to supplement fluoride and enhance fluoride-based nucleation activity, followed by remineralization propelled by salivary and dietary phosphate and calcium [19]. In addition, fTCP shares the same compartment as fluoride to ensure the optimal delivery of both fluoride and fTCP [19]. This suggests that fTCP and fluoride independently affect remineralization. Therefore, fTCP can synchronize with fluoride to provide better efficacy compared with using fluoride alone; this presents an avenue to extend the therapeutic effects of fluoride and improve dental health benefits [19, 22].

**Fig. 7:**
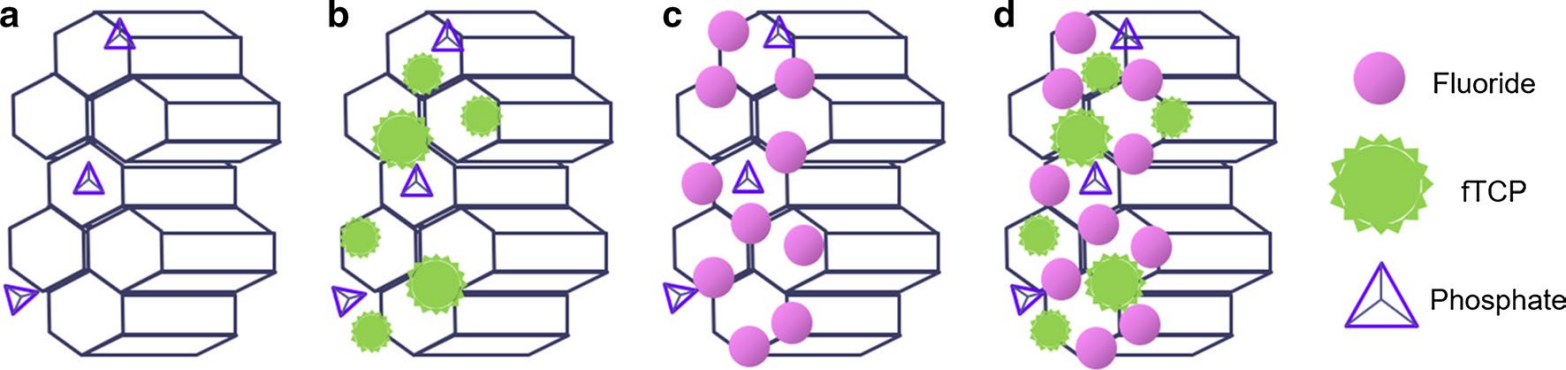
Illustrations depicting the remineralization phenomena occurring when enamel subsurface lesions are treated with fTCP − F − (**a**), fTCP + F − (**b**), fTCP − F + (**c**), and fTCP + F + (**d**) in pH-cycling conditions

The representative cross-sectional SEM micrographs indicated variations in the enamel structure among the groups (Fig. 6). The particles deposited on the enamel rods in the fTCP specimens could comprise calcium phosphate or calcium fluoride. In a previous study, the combination of fTCP and fluoride produced relatively large, densely packed crystals compared with the smaller and/or less dense crystals in the fluoride-free or fluoride-control specimens [19]. fTCP delivers calcium and phosphate ions similar to those of the enamel framework, and this delivery depends on fTCP concentration [19, 24]. Taken together, the findings of these studies, including the present study, show that fTCP promotes the uptake of ions, including fluoride ions, into the enamel in a unique manner (Fig. 7). The null hypothesis of this study was rejected because the application of fTCP + F + on the enamel subsurface lesions increased the potential for remineralization. The application of fTCP + F + after tooth cavity preparation or as a coating on WSLs or cracks may aid in the prevention and reduction of secondary caries development, prevention of caries progression, and protection of tooth structures. Further studies using fTCP + F + are warranted to elucidate the mechanism by which remineralization of enamel subsurface lesions occurs. fTCP supplementation does not appear to accelerate the kinetics of fluoride; however, it may promote the uptake of ions, the nature of which depends on lesion type, fluoride concentration, and fTCP composition, resulting in the production of a stronger and more acid-resistant mineral compared with fluoride alone [19].

## Conclusions

In the present study, increased remineralization of artificial enamel subsurface lesions was observed in samples exposed to experimental toothpastes containing fTCP and fluoride. Furthermore, the combination of fTCP and fluoride appears to act independently on the remineralization of enamel subsurface lesions.

## Data Availability

All materials described in this manuscript, including all relevant raw data, will be freely available to any scientist wishing to use them for non-commercial purposes, without breaching participant confidentiality. The data of this research is available from Hidenori Hamba (corresponding author).

## Abbreviations

fTCP: Functionalized tricalcium phosphate
TCP: Tricalcium phosphate
μCT: Micro-focus X-ray computed tomography
MD: Mineral density
SEM: Scanning electron microscopy
WSL: White spot lesion
β-TCP: Beta-tricalcium phosphate
HAp: Hydroxyapatite
%R: Percentage of remineralization
